# Opioid-induced respiratory depression and risk factors in a tertiary hospital: A retrospective study

**DOI:** 10.1016/j.jsps.2022.06.004

**Published:** 2022-06-13

**Authors:** Nabil A. Almouaalamy, Majed Alshamrani, Waleed K. Alnejadi, Ziyad M. Alharbi, Faisal M. Aldosari, Enad F. Alsulimani, Saif A. Saif, Mohammed K. Aldawsari

**Affiliations:** a Oncology Department, Princess Noorah Oncology Center, King Abdulaziz Medical City, Ministry of National Guard-Health Affairs, Jeddah, Saudi Arabia; b King Abdullah International Medical Research Centre, Jeddah, Saudi Arabia; c College of Medicine, King Saud bin Abdulaziz University for Health Sciences, Jeddah, Saudi Arabia; d Pharmaceutical Care Services, King Abdulaziz Medical City, Ministry of National Guard-Health Affairs, Jeddah, Saudi Arabia

**Keywords:** Respiratory depression, Cancer pain, Opioids

## Abstract

**Background:**

Opioids are potent analgesics used for the treatment of moderate to severe acute and chronic cancer and non-cancer pain. However, opioid usage may be limited by negative side effects, such as potentially life-threatening respiratory depression.

**Objectives:**

The aim of our study is to investigate the prevalence of opioid-induced respiratory depression (OIRD) and its predictors at King Abdulaziz Medical City in Jeddah (KAMC-JD).

**Method:**

This is a retrospective cross-sectional (chart review) study conducted from January 1, 2016, to December 31, 2020.

**Results:**

A total of 15,753 patients received opioids during admission to KAMC-JD, and only 144 (0.915%) of them received naloxone from January 1, 2016 to December 31, 2020. Only 91 patients (0.57%) developed opioid-induced respiratory depression (OIRD), which was more frequently reported among young and middle-aged adults. OIRD was significantly associated with receiving a daily morphine milligram equivalent (MME) dose of ≥150 MME and with having a low urea concentration at the baseline and at admission under surgery. Also, fentanyl use remained a significant risk factor for OIRD.

**Conclusion:**

In conclusion, monitoring patient receiving opioids with a daily MME dose of ≥150 MME, prescribed Fentanyl, low urea concentration at the baseline, and patients’ admissions to the surgery department may mitigate the risk of developing OIRD.

## 1 Introduction

Opioids are potent analgesics used for the treatment of moderate to severe acute and chronic cancer and non-cancer pain. However, opioids have negative side effects that may limit their usage (Gupta and Atcheson, 2013). The detection and management of adverse drug events (ADEs) and medication errors due to opioids are essential for the proper use of controlled medicines (American Society of Anesthesiologists Task Force on Neuraxial Opioids and the American Society of Regional Anesthesia and Pain Medicine, 2016). Opioid analgesics have several side effects, of which respiratory depression is a potentially life-threatening ADE (American Society of Anesthesiologists Task Force on Neuraxial Opioids and the American Society of Regional Anesthesia and Pain Medicine, 2016). Opioid-induced respiratory depression (OIRD) is defined as (1) reduced respiratory rate (e.g., to <10 breath/min), (2) reduced oxygen saturation (e.g., arterial oxygen saturation <90%), or (3) hypercapnia/hypercarbia (e.g., arterial carbon dioxide tension more than 50 mmHg) (American Society of Anesthesiologists Task Force on Neuraxial Opioids and the American Society of Regional Anesthesia and Pain Medicine, 2016). Previous studies have demonstrated that the risk factors for OIRD include opioid overdose, advanced age, sleep apnea, chronic obstructive pulmonary disease, congestive heart failure, renal failure, and hemodialysis/peritoneal dialysis (Southern and Read, 1994, Cepeda et al., 2003, Ostermeier et al., 1997, Taylor et al., 2005, Ravenscroft and Schneider, 2000, Baxter, 1994, Izumi et al., 2012). However, to date, no nationwide data have been collected for OIRD in cancer and non-cancer patients in Saudi Arabia. Because respiratory depression is a rare ADE associated with opioids, the implementation phase of epidemiologic research is difficult. The aim of our study is to investigate the prevalence of OIRD and its predictors in our institute and to recommend ways to improve the prescription of opioids for our patients.

## 2 Materials and methods

This a retrospective cross-sectional study (chart review) study conducted from January 1, 2016 to December 31, 2020, to investigate the prevalence of OIRD and its predictors at King Abdulaziz Medical City in Jeddah (KAMC-JD). The study design was approved by the [BLINDED FOR REVIEW], and informed consent was waived due to the study design. KAMC-JD is a tertiary hospital that includes Princess Noorah Oncology Center and King Faisal Cardiac Center with 558 functional beds within the Makkah region (western region) of the Kingdom of Saudi Arabia. We included all the adult patients (18 years and above) who were admitted into KAMC-JD and who received naloxone as a result of opioid side effects during the study period. The exclusion criteria were incomplete charts and privacy requests.

### 2.1 Data collection

The source of data was the BESTCare Health Information System (HIS). The following data were retrieved from the BESTCare HIS: age, sex, marital status, area of residence, code status, admitting diagnosis, admitting service, length of stay (LOS) for each admission, comorbidities, opioid name and dose, and the reason for giving the patient naloxone.

To standardize all the different prescribed opioid doses, they were all converted to morphine milligram equivalents (MME) per day, and to accomplish that, we used the following formula: Opioid dosage × the number of doses per day × the MME conversion factor = MME. Every opioid conversion factor was based on its potency using the Centers for Disease Control (CDC) conversion table (CDC Opioid Conversion Guide https://www.cdc.gov/drugoverdose/pdf/calculating_total_daily_dose-a.pdf)).

### 2.2 Definitions

In the present study, OIRD was defined as (1) reduced respiratory rate (e.g., to<10 breath/min), (2) reduced oxygen saturation (e.g., arterial oxygen saturation<90%), or (3) hypercapnia/hypercarbia (e.g., arterial carbon dioxide tension more than 50 mmHg (American Society of Anesthesiologists Task Force on Neuraxial Opioids and the American Society of Regional Anesthesia and Pain Medicine, 2016). The weak opioids included tramadol, meperidine, and codeine, whereas the strong opioids included morphine, fentanyl, hydromorphone, methadone, oxycodone, and hydrocodone.

### 2.3 Statistical analysis

We presented categorical data as frequencies and percentages, whereas the numerical data were expressed as medians and interquartile ranges (IQRs). The statistical tests of univariate associations included the chi-squared test for categorical variables and the Mann-Whitney test for continuous variables. These tests were applied to assess the factors associated with naloxone prescription and OIRD (the primary outcomes). Furthermore, since OIRD is considered an adverse event for opioids, the reporting odds ratio (ROR) was calculated for the suspected individual medications. The analysis was performed by constructing a cross-tabulation as follows: ROR = (a/b)/(c/d), where *a*: the number of cases with OIRD after using a suspected opioid medication, *b*: the number of cases with OIRD after using other opioid medications, *c*: the number of cases with other adverse events after using a suspected opioid medication, and *d*: the number of cases with other adverse events after using other opioid medications. To account for potential bias, the Haldane-Anscombe correction was performed by adding ½ to cell counts with zero (Haldane, 1956). The corresponding 95% confidence intervals (95% CIs) were also expressed in the ROR analysis. A suspected opioid medication was considered to be associated with OIRD if the ROR and the lower limits of 95% CIs exceeded 1.

Independent associations were further tested via fitting logistic regression models (using the [enter] method), considering the primary outcomes as dependent variables and the significantly associated factors from the univariate analysis as independent variables. Furthermore, the analysis was adjusted for patients’ demographic and clinical characteristics. The results of the regression analyses were expressed as adjusted odds ratios (aORs) and their respective 95% CIs. A statistical analysis was performed using the Statistical Package for Social Sciences version 26.0 (SPSS Inc., Chicago, IL, USA) and the *epiR* package in R software (R i386 version 4.0.0).

## 3 Results

### 3.1 Demographic and clinical characteristics of patients

**Fig. 1:**
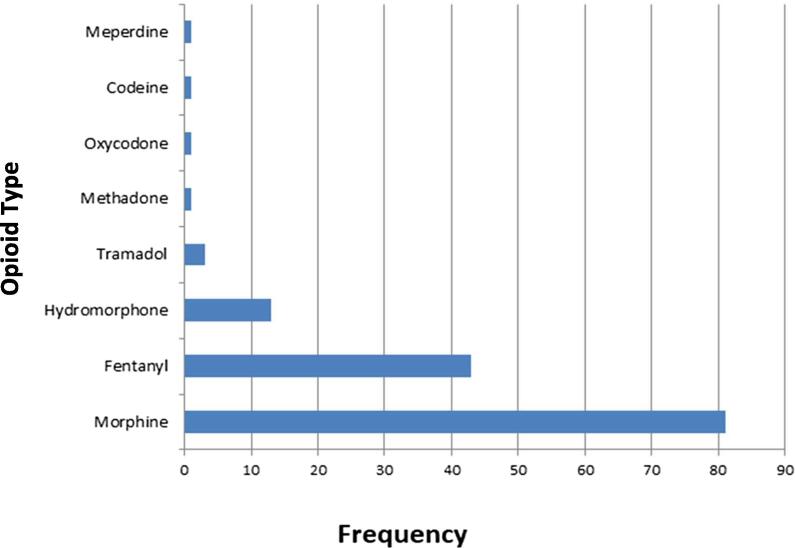
The frequency of opioid prescribing among the patients (n = 144).

The data were retrieved for a total of 15,735 patients (MME 23.07 MME) who received opioids, but only 144 patients (0.915%) received naloxone from January 1, 2016 to December 31, 2020. An analysis of the 144 patients revealed that more than half of the patients were females (59%) and that the majority of them were married (82.6%). Additionally, 43.75% of patients were ≥ 65 years old. The detailed descriptive statistics of the other demographic and clinical characteristics are listed in Table 1. Focusing on the patterns of opioids prescription, a total of nine opioid medications was prescribed to all the patients; these predominantly included morphine (56%) and fentanyl (29.9%, Fig. 1).

**Table 1:** Demographic and clinical characteristics of the patients and the factors associated with OIRD among them (n = 144).

Parameter	Category	OIRD		p
		No (n = 53)	Yes (n = 91)	
Age categories	18–29	2 (13.33)	13 (86.67)	**0.010**
	30–49	6 (20)	24 (80)	
	50–64	14 (38.89)	22 (61.11)	
	≥ 65	31 (49.21)	32 (50.79)	
Gender	Female	33 (38.82)	52 (61.18)	0.547
	Male	20 (33.9)	39 (66.1)	
Marital status	Single	3 (21.43)	11 (78.57)	0.532
	Married	46 (38.66)	73 (61.34)	
	Widowed	1 (33.33)	2 (66.67)	
	Divorced	0 (0)	1 (100)	
BMI (kg/m^2^)	Median (IQR)	27.1 (21.8–33.5)	29.3 (23.6–34.9)	0.234
Body temperature (°C)	Median (IQR)	36.9 (36.7–37.0)	36.8 (36.6–36.9)	0.126
Respiratory rate	Median (IQR)	20.0 (19.0–21.0)	20.0 (19.0–20.0)	0.906
Heart Rate	Median (IQR)	90.0 (80.0–112.0)	87.0 (76.0–97.0)	0.075
Systolic blood pressure (mmHg)	Median (IQR)	125.0 (110.5–137.5)	130.0 (110.0–148.0)	0.283
Diastolic blood pressure (mmHg)	Median (IQR)	66.0 (60.0–76.0)	69.0 (59.0–80.0)	0.513
Urea	Median (IQR)	7.3 (4.2–16.1)	5.0 (3.4–8.0)	**0.018**
Creatine value	Median (IQR)	94.0 (64.5–148.5)	69.0 (60.5–114.8)	0.124
Length of stay in each admission (d)	Median (IQR)	6 (3–14)	5 (4–8)	0.599
Benzodiazepine use	No	45 (35.4)	82 (64.6)	0.351
	Yes	8 (47.1)	9 (52.9)	
Specialty on hospital admission	Medicine	19 (55.9)	15 (44.1)	**< 0.0001**
	Oncology	22 (55.0)	18 (45.0)	
	Surgery	12 (17.1)	58 (82.9)	
Equivalent oral morphine dose	< 50 MME	29 (50)	29 (50)	**< 0.0001**
	50 to < 150 MME	12 (66.7)	6 (33.3)	
	≥ 150 MME	12 (17.6)	56 (82.4)	

MME: morphine milligram equivalents.

OIRD was significantly associated with age in young and middle-aged adults, with receiving a daily morphine milligram equivalent (MME) dose of ≥ 150 MME, with having a low urea concentration at the baseline and at admission under surgery.

### 3.2 The predictors of ORID and associated actors

**Fig. 2:**
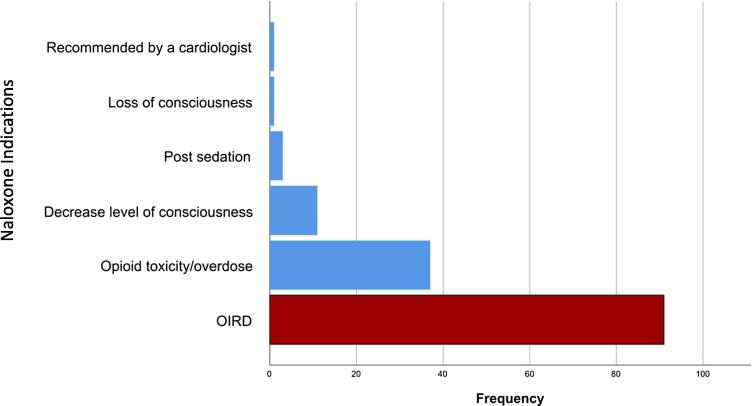
The indication of naloxone prescribing among the patients (n = 144).

In general, 91 patients experienced ORID, which represented 63.2% of patients for whom naloxone was exclusively reported **(**Fig. 2**)**. The greater proportions of patients aged 18–29 and 30–49 years had experienced OIRD (86.7% and 80%, respectively) compared to older patients (61.1% for 50–64 years and 50.8% for ≥ 65, respectively, p = 0.010). Additionally, urea concentrations were significantly lower among patients with OIRD than those without OIRD (median [IQR] = 7.3 [4.2 to 16.1] for OIRD versus 5.0 [3.4 to 8.0] for non-ORID, p = 0.018). Of note, a significantly higher proportion of the patients with OIRD had received the highest daily doses of opioids (≥150 MME to 82.4% of patients) compared to those who had received 50 to < 150 MME (33.3%) and < 50 MME (50.0%, p < 0.0001), and they were admitted to the surgery department (82.9%) compared to those admitted to the oncology and medical departments (45.0% and 44.1%, respectively, p < 0.0001, Table 1).

To assess the independent association between daily MME dose and OIRD cases that necessitated naloxone use, we fitted a logistic regression model with the OIRD status as a dependent variable and the significantly associated factors from the univariate analysis as independent variables (Table 2). The analysis revealed that the specialty of admission was the sole independent risk factor of ORID, where the admission to the surgery department was independently associated with ORID (aOR = 3.03, 95% CI, 1.06 to 8.70, *p* = 0.039), but that was not the case with admissions to the oncology department.

**Table 2:** The results of the logistic regression analysis for the risk factors of OIRD among the patients (n = 144).

Parameter	Category	OR	95% CI	p
Age	18–29	Ref		
	30–49	1.41	0.20–9.91	0.729
	50–64	0.72	0.11–4.69	0.732
	≥ 65	0.45	0.08–2.70	0.383
Equivalent oral morphine dose	<50 MME	Ref		
	50 to < 150 MME	0.45	0.13–1.54	0.206
	≥150 MME	2.42	0.92–6.32	0.072
Urea concentration	Numerical	0.98	0.93–1.03	0.413
Specialty on hospital admission	Oncology	Ref		
	Medicine	1.06	0.38–2.97	0.909
	Surgery	3.03	1.06–8.70	**0.039**

MME: morphine milligram equivalents.

OIRD was significantly associated with admission under surgery.

Focusing on the individual medications, out of the nine opioids that were prescribed to patients, only fentanyl showed a large ROR (3.37, 95% 1.51 to 7.53, *p* = 0.002, Table 3), indicating an increased risk for OIRD. On the adjustment for potential confounders, fentanyl use remained a significant risk factor for OIRD cases that necessitated naloxone prescription (aOR = 3.53, 95 %CI, 1.24 to 10.07, *p* = 0.018). Notably, naloxone use for OIRD did not differ by the prescription of weak or strong opioids, as indicated by the small RORs (Table 3).

**Table 3:** The number of reported cases and the reporting odds ratios of opioid-induced respiratory depression among the patients (n = 144).

Opioid	Cases	ROR	95% CI	*p*
Weak opioids	5	0.33	0.10–1.06	0.053
Strong opioids	89	3.63	0.64–20.55	0.121
Morphine	49	0.83	0.42–1.64	0.589
Fentanyl	40	3.37	1.51–7.53	**0.002**
Hydromorphone	5	0.28	0.09–0.9	0.025
Tramadol	3	0.22	0.06–0.91	0.024
Methadone	0	0.11	0.01–2.39	0.282
Oxycodone	0	0.11	0.01–2.39	0.282
Meperidine	1	1.77	0.07–44.32	0.531
Codeine	1	0.58	0.04–9.43	0.697
Hydrocodone	1	1.77	0.07–44.32	0.531

ROR: reporting odds ratios.

OIRD was significantly associated with receiving Fentanyl.

## 4 Discussion

In Saudi Arabia, drug overdoses have been reported elsewhere in the literature among small cohorts over the past few decades (Elfawal, 1999). A dearth of data exists regarding the patterns of adverse events for opioid prescription overdose on the national level (Ministry of Health – Saudi Arabia, 2018), and little is known about the prevalence of OIRD cases that necessitate naloxone prescription. In the present study, we investigate the prevalence of opioid-induced respiratory depression (OIRD) and its predictors among patients who were prescribed opioids. The results showed that OIRD occurred in 0.57% of our total sample, also naloxone was prescribed to the (0.915%) patients receiving opioids during the study period and almost two-third of them (63.2%) due to OIRD. Additionally, ORID was more frequently reported among young and middle-aged adults, and it was significantly associated with receiving a daily MME dose of ≥ 150 MME, a low urea concentration at the baseline, and patients’ admissions to the surgery department. Nonetheless, the latter was the sole predictor of ORID.

Because respiratory adverse events are rarely reported, we used the ROR approach to investigate the risk of OIRD. Such a statistical method has proven effective to provide an initial signal for relative risk and demonstrate the magnitude of the adverse events with minimal possible bias (Rothman et al., 2004). The ROR analysis indicated a significant risk of OIRD with fentanyl prescription. In a recent investigation employing a similar approach among cancer patients in Japan, Sugawara et al. (2019) showed that the RORs and their respective 95% CIs exceeded 1 for eight different opioids, with the highest values reported for fentanyl, morphine, and oxycodone. The variation in the findings may be attributable to the small sample size under investigation in our study (n = 144) compared to the Japanese study (n = 1227). Therefore, future large-sized, nation-wide investigations are needed in Saudi Arabia to emphasize the influence of each opioid medication on the risk of OIRD.

However, we showed that fentanyl use was a significant risk factor for ORID regardless of all the demographic and clinical variables of patients, which was consistent with other findings in the literature (Hill et al., 2020, Boom et al., 2012, Kiyatkin, 2019). Only a small amount of fentanyl is required to induce profound effects; therefore, a small error in the administered dosage may cause severe adverse events. Moreover, fentanyl rapidly penetrates the brain, and several reports have indicated that naloxone may be less sensitive to reverse fentanyl-induced respiratory depression than other opioids (Rzasa Lynn and Galinkin, 2018, Fairbairn et al., 2017). Interestingly, recent in vivo studies have demonstrated that fentanyl-induced apnea was prevented and reversed via the microinjection of a selective antagonist of the mu opioid receptor into the pontine Kölliker-Fuse/parabrachial complex (KF/PB) (Saunders and Levitt 2020), which plays a pivotal role in the control of respiration, chemosensory reflex control, and eupneic respiratory pattern formation (Dhingra et al., 2019, Zuperku et al., 2017). The data underline the importance of prescribing fentanyl to selected patients who are clinically less prone to develop ORID.

In the present study, OIRD was significantly associated with receiving high doses of opioids (≥150 MME) in the univariate analysis, but the correlation was no longer significant in the adjusted multivariate analysis. Although no consensus has been established regarding the cutoff point at which a distinct dose of opioids can lead to death, the recent Canadian Guidelines for Opioid Therapy and Chronic Noncancer Pain (Rothman et al., 2004) indicated a “watchful” dose of ≥200 MME based on the published studies in the literature and expert opinions. However, the officially recommended dose is restricted to <90 mg MME (Busse et al., 2017). Furthermore, In the most recent CDC’s Opioid Prescribing Guidelines (Dowell et al., 2016), it has been recommended that clinicians should consider naloxone prescription for patients who are at an increased risk of overdose, including those with a history of overdose, concurrent benzodiazepine use, and high opioid dosages (≥50 MME/day). In a recent observational study of U.S. adults (n = 23,778), Lin et al. (Lin et al., 2020) showed that high daily MME (50 to <90 MME), concurrent benzodiazepine usage, and a confirmed diagnosis of opioid use disorder were all independently associated with naloxone. However, the patterns of co-prescription of naloxone and opioids are generally in agreement with the recent recommendations of large-sized studies and regional regulations in the United States (Luu et al., 2019, Sohn et al., 2020, Katzman et al., 2020).

Importantly, the present study revealed that patients admitted to surgical departments were more likely to experience ORID. The risk of OIRD in the postoperative period has been studied in multiple articles (Dahan et al., 2010, Weingarten et al., 2016, Rosenfeld et al., 2016). The incidence of OIRD has generally ranged between 2% and 37% after surgery, and the majority of cases have occurred within the first six hours after surgical procedures (Dahan et al., 2010, Weingarten et al., 2016, Rosenfeld et al., 2016, Weingarten et al., 2015a, Weingarten et al., 2015b). Furthermore, OIRD is mostly mediated by the additive effects of opioids given to counteract postoperative pain (as well as residual anesthetic drugs) (Gupta et al., 2018). The most frequently cited predictors of naloxone administration to treat postoperative respiratory depression include the history of obstructive sleep apnea, obesity, cardiac diseases, and chronic obstructive pulmonary disease (Dahan et al., 2010, Weingarten et al., 2016, Rosenfeld et al., 2016, Weingarten et al., 2015b). We could not indicate the risk factors for OIRD postoperatively due to the small sample size of patients admitted to surgical wards in this study. The limitations of this study are that it relies on the small sample size that might mediate the lack of statistical relationships and group-based differences. Additionally, the retrospective design of our study might have limited the ability to obtain causal relationships between the different variables. Also, since this study was based on experiences from one center, its generalizability to other settings could be limited.

## 5 Conclusion

In conclusion, OIRD occurred in 0.57% of our total sample, admission to a surgical department was a significant risk factor for OIRD, and fentanyl was the sole opioid that was significantly associated with OIRD regardless of the demographic and clinical characteristics of patients. Our results showed that monitoring patient receiving opioids with a daily MME dose of ≥150 MME, prescribed Fentanyl, low urea concentration at the baseline, and patients’ admissions to the surgery department may mitigate the risk of developing OIRD.

### 5.1 Limitations

The present study has some limitations. The small sample size might mediate the lack of statistical relationships and group-based differences, especially for the demographic and clinical correlates of naloxone prescription. Additionally, the retrospective design of our study might have limited the ability to obtain causal relationships between the different variables.

## Declaration of Competing Interest

The authors declare that they have no known competing financial interests or personal relationships that could have appeared to influence the work reported in this paper.
